# Supplementary material for: Host-targeted niclosamide inhibits C. difficile virulence and prevents disease in mice without disrupting the gut microbiota
Source: Nat Commun. 2018 Dec 7;9:5233. doi: 10.1038/s41467-018-07705-w (PMC6286312; doi:10.1038/s41467-018-07705-w)
Supplement: Supplementary file 1 — Supplementary info [file 41467_2018_7705_MOESM1_ESM.pdf]

Supplementary Information

Host-targeted niclosamide inhibits *C. difficile* virulence and prevents disease pathogenesis in mice without disrupting the gut microbiota

Tam et al.

Drug	% inhibition of cell rounding
**QUINACRINE HYDROCHLORIDE**	98, 82, 80
Carvedilol	92
EBSELEN	82
**AMODIAQUINE DIHYDROCHLORIDE**	77
Nocodazole	74
Rotenone	73
CHLORTETRACYCLINE HYDROCHLORIDE	72
BENZETHONIUM CHLORIDE	72
**Niclosamide**	71
5-(N,N-hexamethylene)amiloride	70
MUNDULONE	69
MECLOCYCLINE SULFOSALICYLATE	68
CHICAGO SKY BLUE	67
CISPLATIN	66
Bay 11-7085	65
ZM 39923 hydrochloride	65
CETYLPYRIDINIUM CHLORIDE	63
Hexahydro-sila-difenidol hydrochloride, p-fluoro analog	62
Bay 11-7082	62
**CHLOROQUINE DIPHOSPHATE CRYSTALLINE**	61
3-(1H-Imidazol-4-yl)propyl di(p-fluorophenyl)methyl ether hydrochloride	61
HEXACHLOROPHENE	60
**CLOSANTEL**	60
Pifithrin-mu	59
HYDROQUINONE	58
Vincristine sulfate	57
Loratadine	57
Sanguinarine chloride	56
MONENSIN SODIUM	56
Perphenazine	55
PHENYL AMINOSALICYLATE	55
2,3-DIHYDROXY-4-METHOXY-4'-ETHOXYBENZOPHENONE	55
3-METHOXYCATECHOL	55
Chelerythrine chloride	54
AURIN TRICARBOXYLIC ACID	54
CGP-74514A hydrochloride	54
Emetine dihydrochloride hydrate	53
Cisplatin	53
**DICHLOROPHEN**	53
ONONETIN	52
**TETRAMIZOLE HYDROCHLORIDE**	52
Z-L-Phe chloromethyl ketone	51
AMINACRINE	51
Piceatannol	51
Picropodophyllotoxin	51
**OXIBENDAZOLE**	50
CEAROIN	50
GR 127935 hydrochloride hydrate	50
U-73122	50
AGARIC ACID	50
CARMUSTINE	50
Vinblastine sulfate salt	49
DEOXYSAPPANONE B 7,3'-DIMETHYL ETHER ACETATE	48
EPIGALLOCATECHIN-3-MONOGALLATE	48
Colchicine	46
Supercinnamaldehyde	46
CCG-4986	45
4-Phenyl-3-furoxancarbonitrile	45
SKF 96365	45
**OXYCLOZANIDE**	43

Supplementary Fig. 1: Summary table of hits from the TcdB cell rounding screen

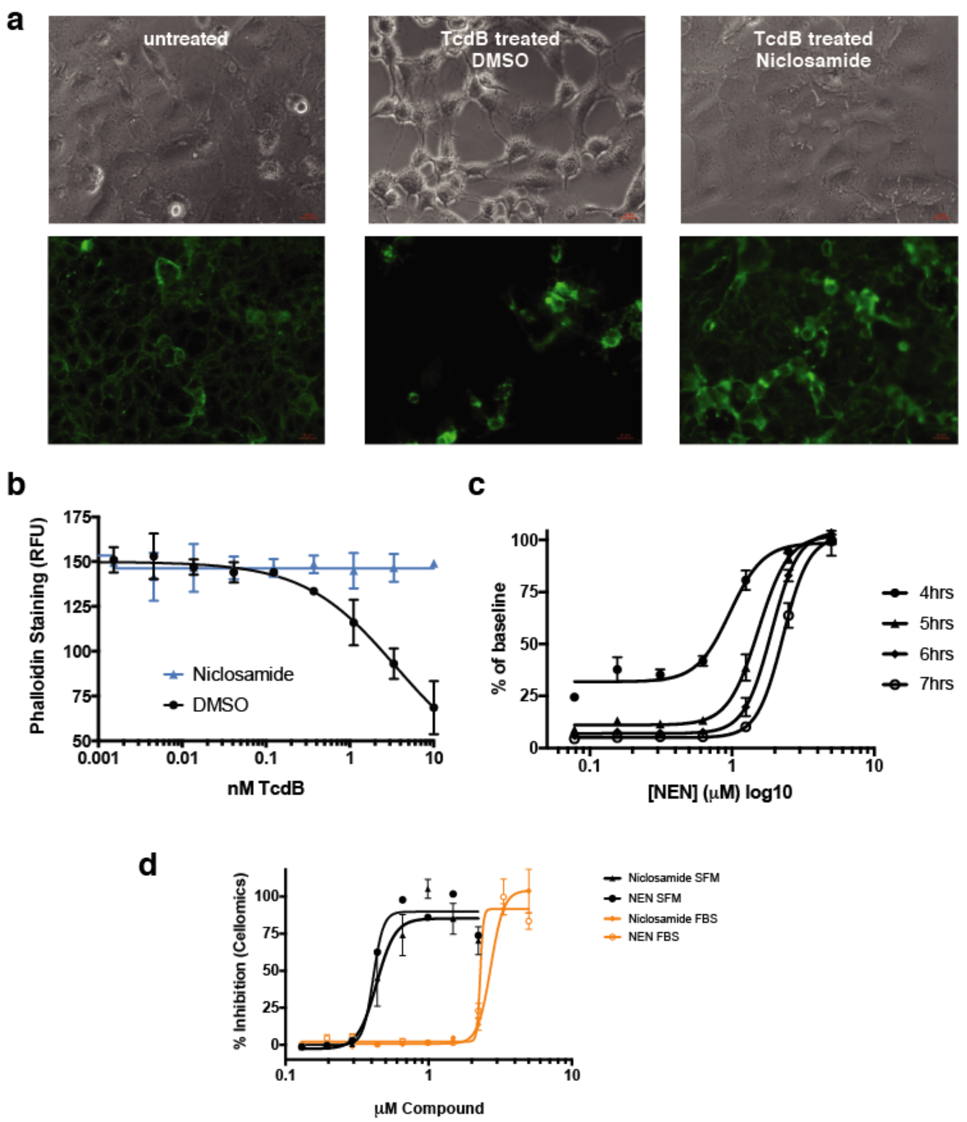

Supplementary Fig. 2: Characterization of niclosamide and NEN. (a) Confluent cultures of Caco2 cells were treated with 10nM TcdB and 1μM Niclosamide or vehicle for 24hrs. Cells were stained with Phalloidin Alexa488, and pictures were taken with a 20X objective using phase contrast or FITC fluorescence filters. (b) Phalloidin staining of cells treated to a serial dilution of TcdB and measured in a fluorescent plate reader (Molecular Devices Spectramax M5e; bottom read, well scan, 9 points/well). Bars represent SEM of 2 replicate wells. (c) Titration of NEN in Caco2 by transepithelial electrical resistance. Caco2 cells were plated on 12-well transwell polyester, 0.4μm pore size plates (Corning), at a density of 100 000 cells/well. Electrical resistance was monitored with a Millicell ERS-2 volt-ohm meter (Millipore). When resistance readings plateaued after 14-21 days, TcdB (5pM final) and test compound were added to the basolateral side. Decline in resistance as a consequence of loss of cell barrier integrity was measured over the next 7 hours and reported as percentage of the baseline value. The IC50s of NEN after 4, 5, 6 and 7 hours were 0.9, 1.5, 1.9 and 2.3 μM, respectively. Bars represent SEM of 3 replicate wells, and the graph is representative of at least 3 experiments. (d) Inhibition of TcdB-induced cell rounding by Niclosamide and NEN in the absence of serum (SFM) and presence of serum (FBS). Values represent mean ± s.e.m.

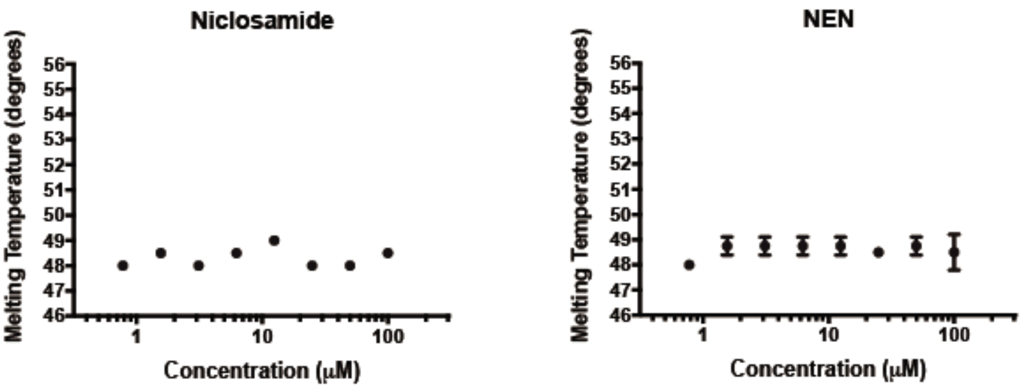

Supplementary Fig. 3: **Real-time protein unfolding assay (DSF)**. Measurement of the thermal melting point of TcdB: 0.05*u*g/*u*L TcdB was combined with 0.3uL test compound in 30*u*L phosphate reaction buffer (KPO4 100mM pH7, 150mM NaCl) in the presence of 5X SYPRO Orange dye (Molecular Probes). Fluorescence as a measure of thermal melting was monitored in a BioRad CFX-96 qPCR thermocycler over a temperature gradient from 15-95 degrees Celcius, in 0.5C° increments. The calculated melting temperatures of TcdB were not affected by up to 100μM Niclosamide or NEN. Values represent mean ± s.e.m.

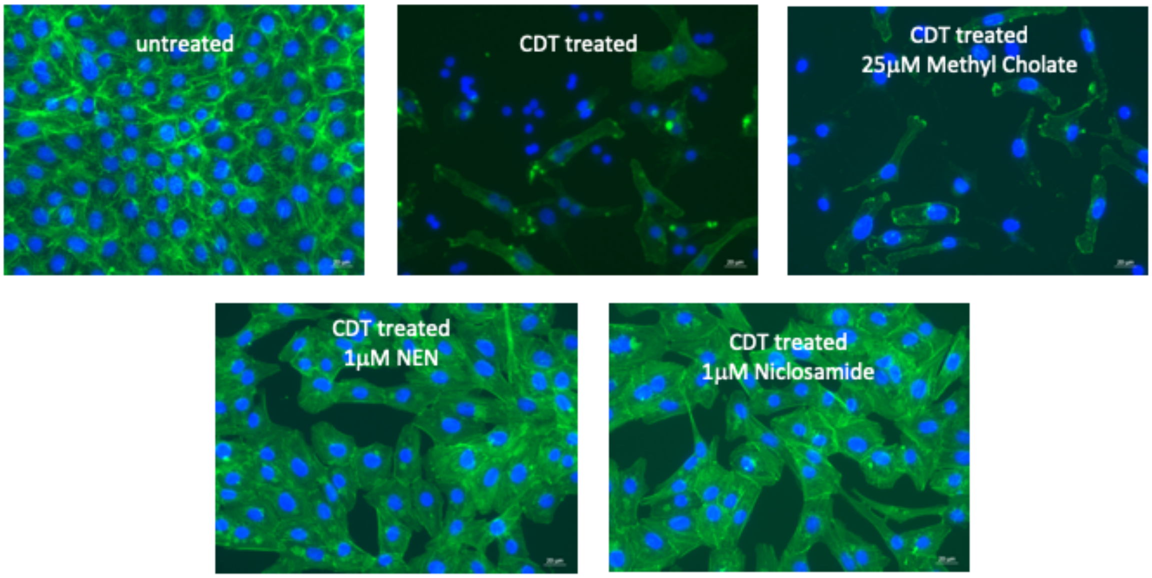

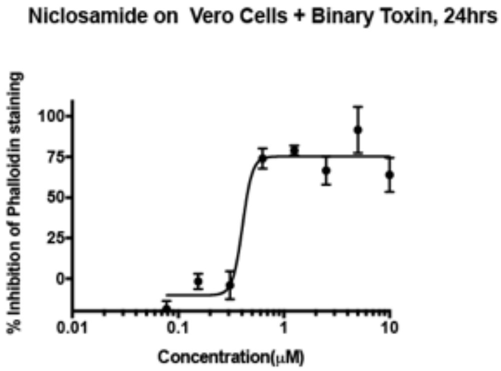

Supplementary Fig. 4: CDT toxicity assay. Vero cells were plated in 96well Cellbind plates at a density of 8000 cells/well (~90% confluent). The next day, compound dilutions and CDT binary toxins (2ug/mL each of A and trypsin-activated B – a kind gift from Merck) in serum free DMEM were added to the plate. After 24hrs, wells were washed with PBS, and the cells were fixed with 4% paraformaldehyde for 15min, permeabilized with 0.25% TX100 for 5min, then stained with Phalloidin Alexa488 (Thermo) for 2hrs before washing and reading Alexa488 fluorescence on a Molecular Devices Spectramax M5e (bottom read, well scan, 9points/well). For microscopy imaging, the cells were stained with 1µM Hoechst for 30min, then combined photographs were taken for each compound at a concentration corresponding to maximum protection from TcdB using a 20X objective and appropriate filter sets for Hoechst and Alexa488 fluorescence (Zeiss). Green represents phalloidin staining while blue represents nuclei. Niclosamide and NEN provided significant protection from loss of phalloidin staining compared to vehicle-treated cells, with an average IC50 of 0.5µM. Values represent mean ± s.e.m (n=3).

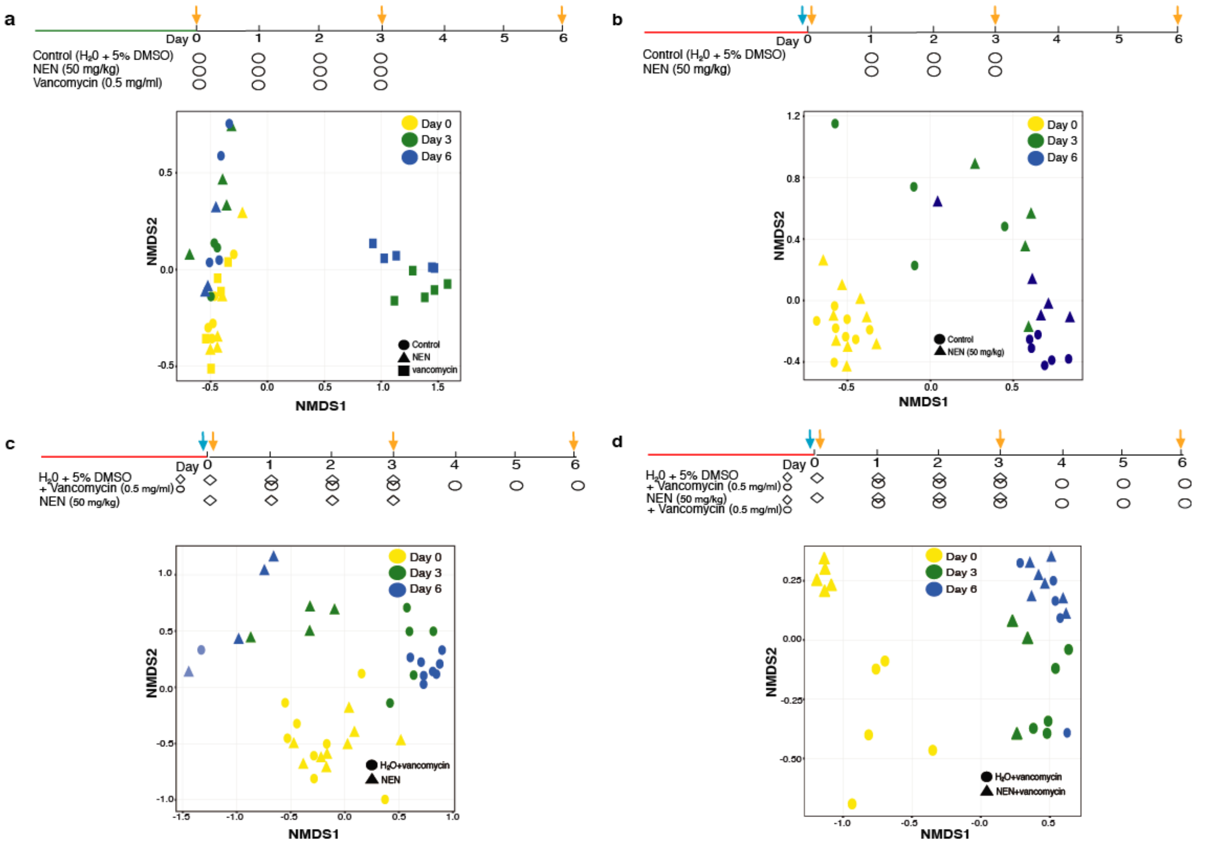

Supplementary Fig. 5: Effect of treatment on the composition and structure of the gut microbiota. Experimental design is shown on top panel (red line indicates antibiotic cocktail treatment six days prior to the *C. difficile* infection for three days, followed by two days of water and clindamycin (30 mg/kg) the day prior to *C. difficile* infection, green line indicates no treatment). Inter-sample analyses were performed using NMDS (nonmetric dimensional scaling) based on Bray-Curtis distance metrics. (**a**) control, NEN, and vancomycin treatment in the absence of *C. difficile* infection; (**b**) control (water with 5% DSMO) and NEN treatment (50 mg/kg); (**c**) water with vancomycin (0.5 mg/ml) and NEN treatment (50 mg/kg); (**d**) water with vancomycin (0.5 mg/ml) and NEN treatment (50 mg/ml) with vancomycin (0.5 mg/ml). First sampling day (day 0) is 4 hours after *C. difficile* infection.

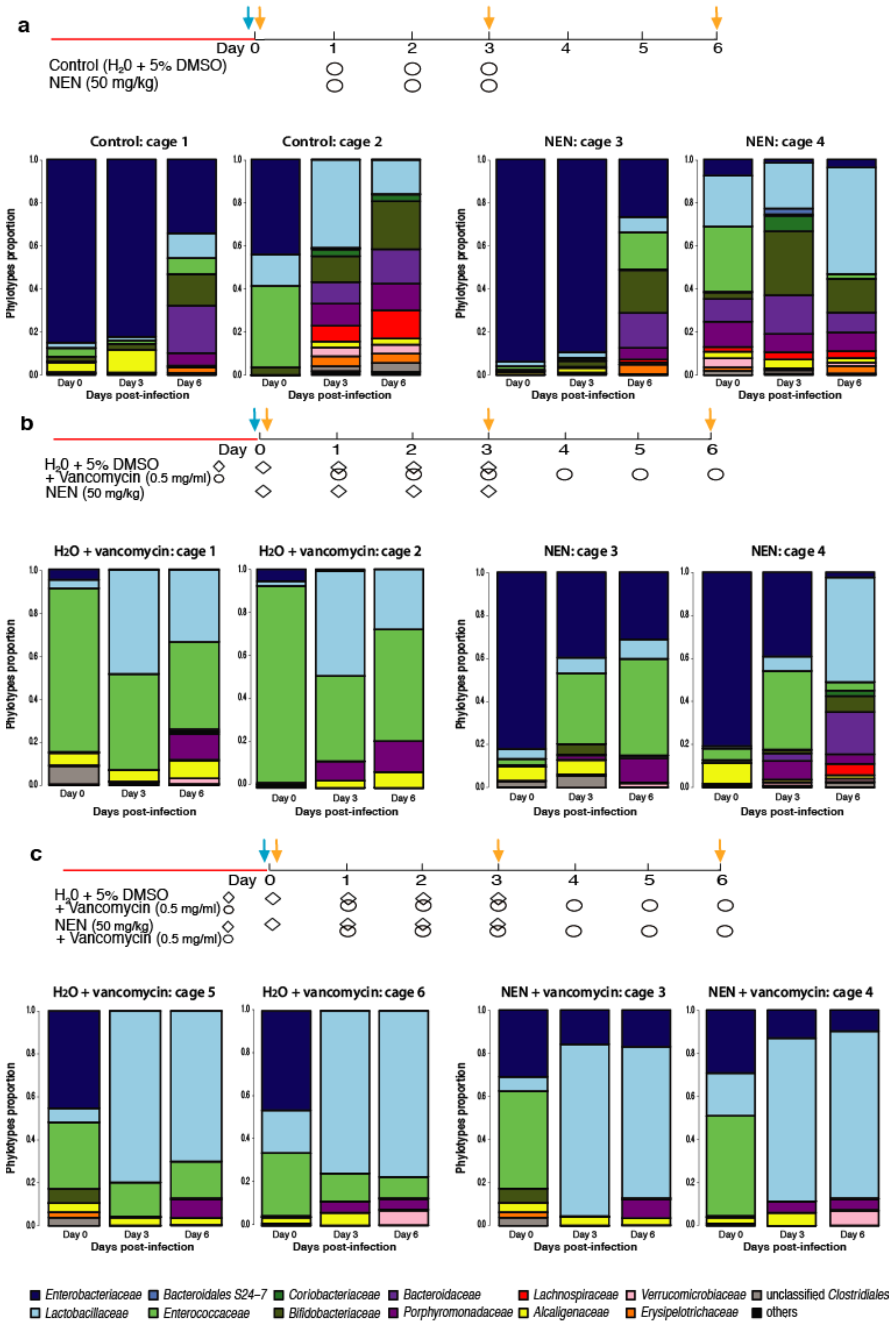

Supplementary Fig. 6: Effect of treatment on the composition of the gut microbiota by cage for each treatment group. Experimental design is shown on top panel (red line indicates antibiotic cocktail treatment six days prior to the *C. difficile* infection for three days, followed by two days of water and clindamycin (30 mg/kg) the day prior to *C. difficile* infection). Each barplot indicates the mean relative abundance of bacterial families with relative abundance >1%. (**a**) control (water with 5% DSMO) and NEN treatment (50 mg/kg). (**b**) water with vancomycin and NEN treatment (50 mg/kg); (**c**) water with vancomycin and NEN treatment (50 mg/ml) with vancomycin (0.5 mg/ml). First sampling day is 4 hours after *C. difficile* infection. Blue arrows represent *C. difficile* infection, while orange arrows represent sampling days.

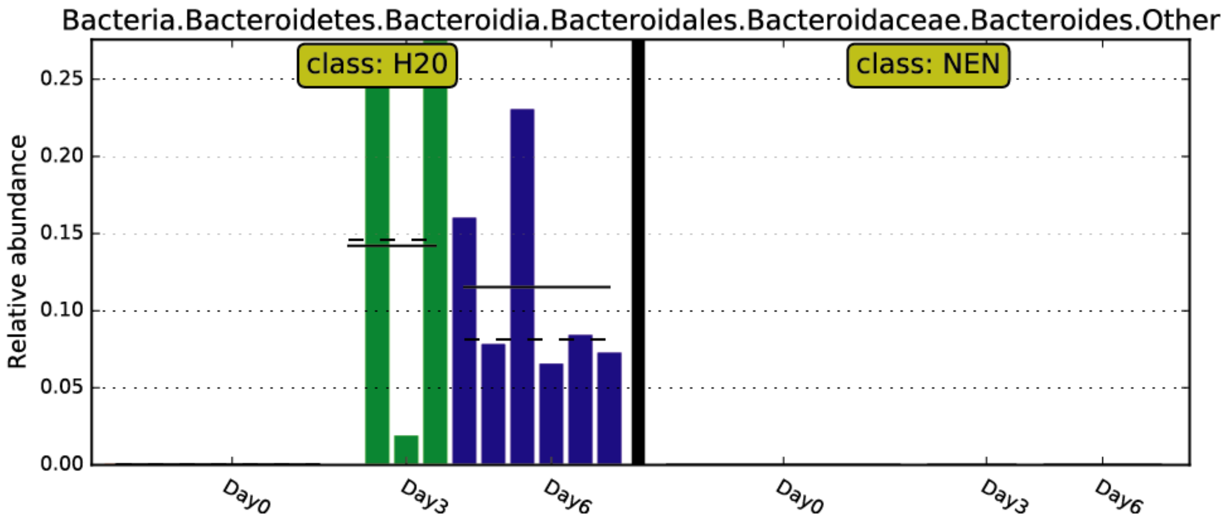

Supplementary Fig. 7: Effect of treatment on a bacterial member of *Bacteriodes*. Bars represent the relative abundance of *Bacteriodes* spp. in each sample. Dotted line represents mean and solid line represents median relative abundance. The alpha value for the non-parametric factorial Kruskal-Wallis (KW) sum-rank test was 0.05 and the threshold for the logarithmic LDA model (43) score for discriminative features was set at 2.0.

Rac1 Western Blots (Raw data)

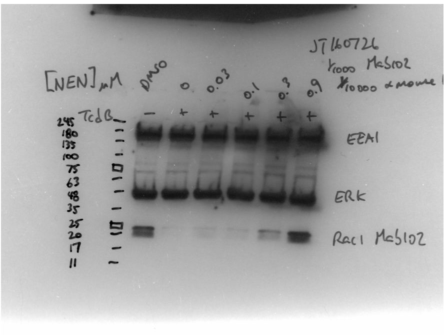

Rac1 Mab102: 1/1000
Anti-ERK, anti-EEA1: 1/2000

Anti-mouse, anti-rabbit HRP: 1/10,000

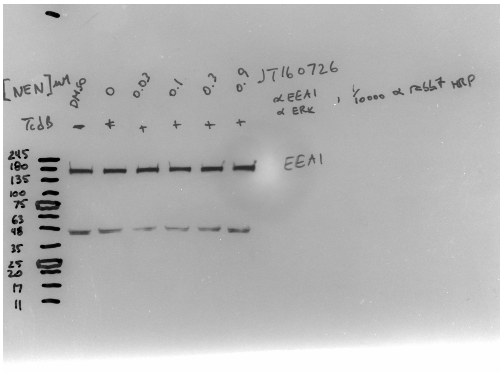

Anti-ERK, anti-EEA1: 1/2000

anti-rabbit HRP: 1/10,000

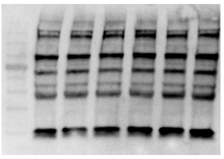

Anti-Rac1 23A8: 1/1000

anti-mouse HRP: 1/10,000

Supplementary Fig. 8: Raw Western blot images for Fig 1a.
