# Supplementary material for: Host-targeted niclosamide inhibits C. difficile virulence and prevents disease in mice without disrupting the gut microbiota
Source: Nat Commun. 2018 Dec 7;9:5233. doi: 10.1038/s41467-018-07705-w (PMC6286312; doi:10.1038/s41467-018-07705-w)
Supplement: Supplementary file 3 — Reporting Summary [file 41467_2018_7705_MOESM3_ESM.pdf]

nature research

Corresponding author(s): Roman A. Melnyk

Reporting Summary

Nature Research wishes to improve the reproducibility of the work that we publish. This form provides structure for consistency and transparency in reporting. For further information on Nature Research policies, see Authors & Referees and the Editorial Policy Checklist.

Statistical parameters

When statistical analyses are reported, confirm that the following items are present in the relevant location (e.g. figure legend, table legend, main text, or Methods section).

n/a	Confirmed	
☐	☒	The exact sample size (*n*) for each experimental group/condition, given as a discrete number and unit of measurement
☐	☒	An indication of whether measurements were taken from distinct samples or whether the same sample was measured repeatedly
☐	☒	The statistical test(s) used AND whether they are one- or two-sided<br>*Only common tests should be described solely by name; describe more complex techniques in the Methods section.*
☒	☐	A description of all covariates tested
☐	☒	A description of any assumptions or corrections, such as tests of normality and adjustment for multiple comparisons
☐	☒	A full description of the statistics including central tendency (e.g. means) or other basic estimates (e.g. regression coefficient) AND variation (e.g. standard deviation) or associated estimates of uncertainty (e.g. confidence intervals)
☐	☒	For null hypothesis testing, the test statistic (e.g. *F*, *t*, *r*) with confidence intervals, effect sizes, degrees of freedom and *P* value noted<br>*Give P values as exact values whenever suitable.*
☒	☐	For Bayesian analysis, information on the choice of priors and Markov chain Monte Carlo settings
☒	☐	For hierarchical and complex designs, identification of the appropriate level for tests and full reporting of outcomes
☒	☐	Estimates of effect sizes (e.g. Cohen's *d*, Pearson's *r*), indicating how they were calculated
☐	☒	Clearly defined error bars<br>*State explicitly what error bars represent (e.g. SD, SE, CI)*

Our web collection on statistics for biologists may be useful.

Software and code

Policy information about availability of computer code

Data collection	No software was used.
Data analysis	The GreenGene database version 13.8 was used for taxonomic classification. Within-sample diversity was estimated using Shannon diversity index. Inter-community comparative analyses were performed using NMDS (nonmetric dimensional scaling) based on Bray-Curtis distance metrics and were plotted using software. package phyloseq. Linear discriminant analysis (LDA) effect size (LEfSe) analysis was applied to identify bacterial phylotypes with relative abundance statistically different between control and NEN (50 mg/kg) treatments. The alpha value for the non-parametric factorial Kruskal-Wallis (KW) sum-rank test was 0.05 and the threshold for the logarithmic LDA model core for discriminative features was set at 2.0.

For manuscripts utilizing custom algorithms or software that are central to the research but not yet described in published literature, software must be made available to editors/reviewers upon request. We strongly encourage code deposition in a community repository (e.g. GitHub). See the Nature Research guidelines for submitting code & software for further information.

Data

Policy information about availability of data

All manuscripts must include a data availability statement. This statement should provide the following information, where applicable:

- Accession codes, unique identifiers, or web links for publicly available datasets
- A list of figures that have associated raw data
- A description of any restrictions on data availability

All 16S rRNA sequence data were deposited in SRA under BioProject PRJNA423011 (SRP128045).

Field-specific reporting

Please select the best fit for your research. If you are not sure, read the appropriate sections before making your selection.

☒ Life sciences ☐ Behavioural & social sciences ☐ Ecological, evolutionary & environmental sciences

For a reference copy of the document with all sections, see nature.com/authors/policies/ReportingSummary-flat.pdf

Life sciences study design

All studies must disclose on these points even when the disclosure is negative.

Sample size	10 to 30 mice per group
Data exclusions	none
Replication	3
Randomization	mice were randomly distributed into different groups
Blinding	yes

Reporting for specific materials, systems and methods

Materials & experimental systems

n/a	Involved in the study	
☐	☒	Unique biological materials
☐	☒	Antibodies
☐	☒	Eukaryotic cell lines
☒	☐	Palaeontology
☐	☒	Animals and other organisms
☒	☐	Human research participants

Methods

n/a	Involved in the study	
☒	☐	ChIP-seq
☒	☐	Flow cytometry
☒	☐	MRI-based neuroimaging

Unique biological materials

Policy information about availability of materials

Obtaining unique materials	C. difficile bacterial strains

Antibodies

Antibodies used	Anti-Rac1 antibody Mab102 was from BD Biosciences (Mississauga, ON); Catalogue number 610650. Total Rac1 levels were measured using mAb23A8 (Abcam); clone 23A8; Cat #Ab33186. Loading controls using anti-EEA1 (catalogue number 07-1820) and anti-ERK1/2 (Catalogue number MABS827) from EMD Millipore. TcdB was detected using anti-TcdB antibody (R&D Systems Catalogue number: AF6246).
Validation	See manufacturers websites.

Eukaryotic cell lines

Policy information about cell lines

Cell line source(s)	CHO-K1, Vero cells, HCT116, CaCo-2, and IMR-90 were all purchased from ATCC (American Type Culture Collection).
Authentication	Cell-lines were authenticated by ATCC.
Mycoplasma contamination	Cell-lines were confirmed by ATCC to be free of mycoplasma contamination.
Commonly misidentified lines (See ICLAC register)	None.

Animals and other organisms

Policy information about studies involving animals; ARRIVE guidelines recommended for reporting animal research

Laboratory animals	Wild-type C57BL/6J, male or female mice, 6 -10 week old.
Wild animals	No
Field-collected samples	No
